# A systematic review of enteral feeding by nasogastric tube in young people with eating disorders

**DOI:** 10.1186/s40337-021-00445-1

**Published:** 2021-07-22

**Authors:** Kristen Hindley, Clare Fenton, Jennifer McIntosh

**Affiliations:** 1 University of Manchester, Manchester, UK; 2 Leeds and York Partnership NHS Foundation Trust, Mill Lodge, 520 Huntington Rd, York, YO32 9QA UK

**Keywords:** Nasogastric, Enteral feeding, Restrictive, Eating disorders, Young people, Adolescents, Inpatient, Anorexia nervosa

## Abstract

**Background:**

Adolescents with severe restrictive eating disorders often require enteral feeding to provide lifesaving treatment.

Nasogastric feeding (NG) is a method of enteral nutrition often used in inpatient settings to treat medical instability, to supplement poor oral intake or to increase nutritional intake. This systematic review sets out to describe current practice of NG in young people with eating disorders.

**Methods:**

A systematic review following PRISMA guidelines was conducted by searching AMED, EMBASE and MEDLINE databases from 2000 to 2020. Inclusion terms were: enteral feeding by nasogastric tube, under 18 years, eating disorders, and primary research. Exclusion terms: psychiatric disorders other than eating disorders; non-primary research; no outcomes specific to NG feeding and participants over 18 years. Titles and abstracts were screened by all authors before reviewing full length articles. Quality assessment, including risk of bias, was conducted by all authors.

**Results:**

Twenty-nine studies met the full criteria. 86% of studies were deemed high or medium risk of bias due to the type of study: 34.4% retrospective cohort and 10.3% RCT; 17.2% were qualitative. Studies identified 1) a wide range of refeeding regimes depending on country, settings, and the reason for initiation; 2) standard practice is to introduce Nasogastric feeds (NG) if medically unstable or oral intake alone is inadequate; 3) NG may enable greater initial weight gain due to increased caloric intake; 4) there are 3 main types of feeding regime: continuous, nocturnal and bolus; 5) complications included nasal irritation, epistaxis, electrolyte disturbance, distress and tube removal; 6) where NG is routinely implemented to increase total calorie intake, length of stay in hospital may be reduced; however where NG is implemented in correlation to severity of symptoms, it may be increased; 7) both medical and psychiatric wards most commonly report using NG in addition to oral intake.

**Conclusions:**

NG feeding is a safe and efficacious method of increasing total calorie intake by either supplementing oral intake or continuously. There are currently no direct comparisons between continuous, nocturnal or bolus regimes, which may be used to direct future treatment for YP with ED.

**Supplementary Information:**

The online version contains supplementary material available at 10.1186/s40337-021-00445-1.

## Plain english summary

Young people with eating disorders often restrict food intake to a degree which is detrimental to their physical health. Depending on the severity of psychiatric and medical symptoms, patients may be admitted to a mental health or medical ward. In the circumstances that their BMI is detrimentally low, a nasogastric (NG) tube may be placed from nose to stomach to pass nutrition. This systematic review sets out to review the current reported evidence of NG in young people. Results have shown that NG feeding is used commonly in the hospital setting to treat medical instability as a result of severe malnourishment, and in the specialist eating disorders (ED) unit due to failure to meet oral intake. NG feeding may be administered through different methods such as continuously, multiple single meals (bolus), or overnight to supplement day-time oral intake. Routine NG feeding may allow greater initial caloric intake, which does not increase risk of medical complications, and may actually increase initial weight gain thus reducing time in hospital. Differences may be due to variable expertise of staff. Side effects are minimal but may include nasal bleeding or irritation, and imbalances in blood electrolytes which can be reduced by providing supplementation.

**Supplementary Information:**

The online version contains supplementary material available at 10.1186/s40337-021-00445-1.

## Background

There are currently over 700,000 individuals in the UK with an eating disorder (ED) [1]. EDs usually manifest prior to adulthood, with an average age of onset of approximately 15 years, although this is decreasing; with new research from NICE demonstrating that incidence in children aged 12 and under had increased between 2005 and 2015 in the UK [2, 3]. Patients with restrictive eating disorders, including anorexia nervosa (AN), bulimia nervosa (BN) and eating disorder not otherwise specified (EDNOS), are predominantly female (91%) and Caucasian (92%), with incidence being approximately 0.014 for females [3]. Compared to other mental illnesses, EDs have a high mortality rate with young people (YP) with anorexia nervosa (AN) on average 6–10 times more likely to die than the general population [4, 5]. Death is often caused by cardiac abnormalities associated with extremely low bodyweight [6]. For this reason, acute medical intervention is often warranted in order to reduce mortality. Nasogastric (NG) feeding use in YP with ED may be used as a lifesaving treatment when patients are physically unwell [7, 8]. However, refeeding is also a critical component to recovery and NG feeding will often be utilised if a young person has been unable to manage oral intake in order to prevent signs of physical unwellness [9, 10].

NG feeding involves a fine bore tube passed via the nasal passage into the stomach in order to administer nutrition. There is a low risk of complications associated with NG feeding if staff receive adequate training and protocols are enforced to ensure that the tube has been passed correctly [11]. Different methods of NG may be utilised safely, with NG feeds often given as large bolus, continuously through a pump or overnight in order to supplement daytime oral intake [12, 13]. Recent guidance from the British Dietetic Association [14] for NG feeding under restraint advised 1–2 bolus feeds per day even in those with high risk of refeeding syndrome (RS); it also concluded further research into this area was required. The National Institute for Clinical Excellence has produced guidance for providing nutrition recommending a graded approach [15]. Neither of these guidelines are specific for children and adolescents.

Most EDs will be treated in an outpatient setting with hospitalisation generally reserved for those with severe malnutrition resulting in physical symptoms such as bradycardia, hypotension or dehydration as set out in the MARSIPAN guidance [16]. Research on NG feeding in YP has tended to focus on the acute refeeding phase in paediatric or psychiatric wards to reduce the risk of RS [17]. RS can manifest as hypophosphatemia (HP), hypomagnesemia, hypokalemia and other electrolyte imbalances that result in cardiac arrhythmias, seizures and in some cases sudden death [18]. During the acute refeeding phase the need for weight restoration must be balanced against the risk of developing RS. Most patients (96%) however present less severely with serum hypophosphataemia and no clinical signs [19]. Although there is a significant body of research into this, the role of NG feeding remains ill-defined [17].

Moreover, for clinicians, there is currently conflicting guidance on how to manage NG feeding in YP with ED, in particular how and when to transition between oral and NG feeding [20, 21]. This has resulted in a variety of NG feeding practices across different settings, with many medical wards tending to provide continuous NG feeds and cease oral intake in order to medically stabilise the patient [20, 22–26]; in contrast mental health wards or specialized eating disorder programs housed on medical wards may be more likely to use syringe bolus feeds to provide food when meals are refused, encouraging oral intake and aiding normalisation of eating [9, 18, 27–31]. In a recent systematic review [32] 9/10 studies in hospitalised ED patients are given continuous or overnight supplemental NG feeding.

Previous reviews [32, 33] have examined use of NG feeding in ED, including the safety and efficacy of NG feeding as well as short-term and long-term outcomes. However, this will be the first systematic review on the use of NG feeding specifically in YP with ED. Due to the anticipated paucity of studies in this area any research where a meaningful conclusion or result can be drawn regarding NG use in YP with ED will be included. This review aims to assess strategies for the use, tolerance and effectiveness of NG feeding in YP with restrictive ED.

## Methods

A comprehensive database search of AMED, EMBASE, APA Psychinfo and MEDLINE was performed with no language restriction from January 2000 to July 2020. Search strategies combined keywords with controlled vocabulary terms (MeSH, Thesaurus); both quantitative and qualitative research were included. The search criteria was peer reviewed by a researcher from the University of York’s Child and Adolescent Mental Health Intervention Centre. References were exported and duplicates were removed using the title and abstract.

### Screening for eligible studies

The full search is available in Appendix 1. The inclusion criteria were: NG feeding, participants under 18 years, eating disorders, published since 2000 and primary research. The outcomes of interest were: Opinions of YP and staff using NG, amount of YP requiring NG, any interventions that impacted on NG feeding, complications of NG feeding, interventions to mitigate the complications, the setting (medical ward, psychiatric ward or outpatient), the NG method and whether this changed when restraint was required. The exclusion criteria included: No ability to discern results specific to NG feeding, mental disorders other than eating disorders being the focus, where the majority of participants are over 18 years or it is impossible to separate results for adults from YP, reviews or other non-primary research and research published before 2000.

**Fig. 1:**
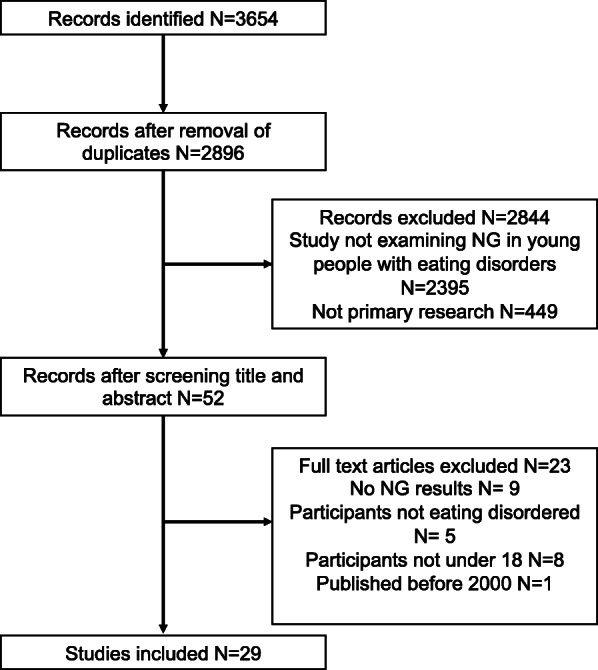
PRISMA Flowchart

Studies published in languages other than English were translated prior to being reviewed. The PRISMA flowchart was used (Fig. 1). Abstracts identified from the initial search were screened in a secondary review process, and full text papers were obtained of those meeting the inclusion criteria or where there was uncertainty. One article published prior to 2000 was included in the full text review due to it requiring translation prior to assessing it against the criteria. Key studies were manually reviewed for additional research, but none were identified that were not already included, 1 eligible study was identified through peer review. There was no disagreement between CF and KH who assessed which studies were included.

Figure 1 displaying PRISMA flowchart of methodology utilised to search databases for this systematic review of enteral feeding in young people with restrictive eating disorders.

### Assessing study quality

There is no validated method to assess the retrospective and qualitative nature of studies included therefore we could not conduct a formal quality assessment or statistical method to evaluate the results. The risk of bias was estimated into high, medium or low using an adapted version of the Agency for Healthcare Research and Quality risk of bias tool as described in Myers [34] which included an assessment of the bias in the selection of participants, sample size, tools used to assess change and whether the study involved blinding. The studies were analysed for risk of bias independently by CF, KH and JM. The risk of bias was deemed to be medium or high (see Additional file 1: Appendix 2) for the majority of the studies included due to the nature of their design, being case series or retrospective cohort studies. Table 1 includes a summary of included studies. Results interpreted from studies with a high risk of bias were removed accordingly, leaving only high quality results and conclusions.

**Table 1:** Summary of Eligible Studies

References	Study Design	Country Set	Time Frame / Follow up years (months)	N total (Female)	Age Range (years)	Setting	Aims	NG Primary/ Secondary Outcome? (Reason for Implementing NG)	Main Outcomes	Risk of Bias
Whitelaw et al., 2010 [9]	Cohort Study (retrospective)	Australia	TF 1	29 (not stated)	12–18	Adolescent Medical Ward	Assess whether more aggressive refeeding leaves patients at greater risk of HP	Secondary (Inadequate oral intake)	HP associated with lower %IBW and lower number of hospital admissions; 15% required NG feeding	Medium
Rocks et al., 2014 [10]	Cross-Sectional Study (prospective)	Australia	TF 0 (3)	17 (n/a)	N/A	Variety of Settings	Describe practices of Australian dietitians in management of AN	Secondary (Inadequate oral intake)	All dietitians stated OR was offered first with supplementation. 82% recommended implementing NG feeding as part of re-feeding process.	Medium
Maginot et al., 2017 [18]	Cohort Study (retrospective)	USA	TF 1	87 (73)	8–20	Medical Behavioural Unit	Safety of higher calorie nutritional rehabilitation protocol (NRP)	Secondary (Inadequate oral intake)	Lower %IBW on admission more important predictor of HP than initial calories. Malnourished patients started on lower calories more likely to have NG tube.	Medium
Paccagnella et al., 2006 [20]	Cohort Study (prospective)	Italy	TF 1	24 (24)	11–32	“Hospital”	Define minimal criteria for “lifesaving” treatment and submit a patient to NG	Secondary (medical instability)	Symptomatology improved the day after NG; is beneficial especially when used for life saving treatment initially	Medium
Silber et al., 2004 [21]	Cohort Study (retrospective)	USA	TF 10	14 (0)	12–18	Adolescent Inpatient Unit	Determine outcomes of supplementing oral refeeding with nocturnal NG supplementation	Primary (Routinely)	Maximum kcals were greater, weight achieved at discharge greater in treatment group compared to oral refeeding only	High
Madden et al., 2015 [22]	RCT (prospective)	Australia/ USA	TF 3	82 (78)	12–18	Paediatric Medical Ward	Long term outcomes of treating to restore weight rather than just to medically stabilise	Secondary (Medical instability)	No difference in hospital days used after initial admission, total fewer days in hospital to achieve medical stability.	Low
Agostino et al., 2013 [23]	Cohort Study (retrospective)	Canada	TF 8 FU 0 (6)	165 (158)	10–18	Paediatric Medical Ward	Difference in LOS between adolescent ED treated with short-term continuous NG feeding vs. managed with lower calorie meals	Primary (Routinely)	LOS reduced in the NG-fed cohort; No significant difference in complications or electrolyte abnormalities (90% NG cohort received prophylactic phosphate).	Medium
Parker et al., 2016 [24]	Cohort Study (retrospective)	Australia	TF 3	167 (152)	14–19	Adolescent ED unit	Weight gain and complications associated with refeeding prescribed greater initial calories	Secondary (Medical instability)	Mean starting intake was 2611.7 kcal/day (58.4 kcal/kg) With inclusion of phosphate supplementation no increased risk of RS.	Medium
Madden et al., 2015 [25]	RCT (prospective)	Australia	TF 1 (9)	78 (74)	12–18	Paediatric ED service	More rapid refeeding protocol promotes initial weight recovery and medical stability.	Primary (Medical instability)	Adequate weight gain and minimal adverse effects were observed. All patients gained weight in week 1 with no cases of HP or RS.	Low
Kezelman et al., 2018 [26]	Cohort (prospective)	Australia	TF 1 (2) FU 8–66 days	31 (31)	15–19	Specialist ED Adolescent medical ward	Explore the relationship between anxiety and weight restoration	Secondary (Medical instability)	All patients received NG initially. No established relationship between changes in anxiety and weight restoration.	Medium
Fuller et al., 2019 [27]	Cross-Sectional Study (prospective)	UK/ Ireland	TF 1	134 (n/a)	n/a	Variety of Settings	Identify common current practice and if specialist ED units are managing AN differently to other inpatient settings	Primary (Inadequate oral intake)	43.3% reported that they were able to facilitate NG feeding; 79% of units providing NG feeding were able to facilitate physical interventions	Medium
Street et al. 2016 [28]	Case Reports (prospective)	England	TF 3 FU 1–2	31 (30)	10–17	Paediatric medical ward	Evaluate joint care ED pathway between CAMHS and paediatric wards	Secondary (Medical Instability)	Time-limited admissions with boundaried-care plans are easier to manage and enjoyed feeling supported by CAMHS	High
Couturier and Mahmood, 2009 [29]	Cohort Study (retrospective)	Canada	TF 2 FU 1	21 (19)	11–17	Psychiatric Inpatient Unit	Understand whether implementing meal support therapy reduced need for NG	Primary(Inadequate oral intake)	Meal support therapy reduces need for NG (66.7 to 11.1% after implementation (*P* < 0.02))	Medium
Falcoski et al.,2020 [30]	Case Series (prospective)	UK	TF 1	3 (2)	11–14	Specialist ED unit	Evaluate new dietetic guidelines for AN in clinical practice	Primary (variable)	Different use of NG feeding to suit individual; use of continuous and single bolus feeds via NG tube	High
O’Connor et al., 2016 [31]	RCT (prospective)	UK	TF 2	36 (34)	10–16	Paediatric medical Ward	Higher calorie refeeding anthropometric outcomes, cardiac and biochemical markers	Secondary (Inadequate oral intake)	Adolescents on high energy intake had greater weight gain. 11% participants required NG feeding for failure to meet 80% oral intake.	Low
Akgul et al., 2016 [35]	Case Series (retrospective)	Turkey	TF 4	13 (0)	11–17	Paediatric Medical Ward	Describe medical, psychiatric, cultural features of adolescent males with an ED	Secondary (Inadequate oral intake)	Male:female increased (3.6:1 F:M); 2/13 given NG due to refusal to eat in hospital	High
Akgul et al., 2016 [36]	Cohort Study (retrospective)	Turkey	TF 6	35 (28)	11–17	Paediatric Medical Ward	Explore paediatric unit where no specific ED unit for to discuss refeeding approaches and goals for discharge	Primary (variable)	Paediatric ward is acceptable where specialist ED inpatient unit not viable; specialist unit better however limited resources	Medium
Nehring et al., 2014 [37]	Cohort Study (retrospective)	Germany	TF 10 FU 1–12	208 (208)	12–18	Psychiatric Inpatient Unit	Short-term and long-term outcomes of treating with EN compared to no EN	Primary (not discussed)	No significant difference in recovery following NG; 34% had NG	Medium
Neiderman et al., 2004 [38]	Case reports (prospective)	England	FU 1	4 (3)	13–16	Adolescent Unit	Report of gastrostomy or jejunostomy use in 4 cases of AN	Secondary (Medical instability)	4/4 patients required NG feeding and progressed to require gastrostomy/jejuonostomy due to complications	High
Robb et al., 2002 [39]	Cohort Study (retrospective)	USA	TF 6	100 (100)	12–18	Paediatric Medical Ward	Compare short-term outcomes of oral vs. supplemental nocturnal nasogastric refeeding	Primary (Routinely)	Weight gain significantly increased in treatment group, no significant difference in length of hospital stay	Medium
Neiderman et al., 2001 [40]	Cross-Sectional Study (retrospective)	UK	TF 1–18	58 (21 patients 37 parents) (19/21)	Patients 9–17 at start of study	Paediatric Medical Ward	Analyse patient and parent views on NG feeding	Primary (not discussed)	71% patients said they did not consent to NG feeding; patients feared weight gain and loss of control over calorie intake	High
Gusella et al., 2017 [41]	Cohort Study (retrospective)	Canada	TF 13 FU 1	46 (43)	9–15	Outpatient ED team	Compare parent led treatment (PIC) to conventional treatment	Secondary (Medical Instability)	PIC had greater increase in %IBW, fewer hospitalisations, shorter admissions, less likely to receive NG feeding	Medium
Madden et al., 2009 [42]	Cross-Sectional Study (prospective)	Australia	TF 3	101 (74)	5–13	Medical Ward and Psychiatric Inpatient Wards	Collect epidemiological data on EO-ED	Secondary (not discussed)	Most were hospitalised (78%), mean duration of hospitalisation was 24.7 days; 58% inpatients NG tube fed.	Medium
van Noort et al., 2018 [43]	Cohort Study (prospective)	Germany	TF 3	120 (120)	9–19	Specialist ED unit	Evaluate characteristics of EO-AN compared with AO-AN.	Secondary (Inadequate oral intake)	NG tube feeding required more in EO-AN than AO-AN; Restrictive more common in EO.	Medium
Strik Lievers et al., 2009 [44]	Cohort Study (prospective)	France	TF 8	213 (213)	12–22	Psychiatric Ward	Clinical variables influencing the length of stay (LOS) of inpatient treatment for AN	Secondary (Medical instability)	Requirement for tube feeding was predictor for LOS (longer) tube feeding required in 27% admissions.	Medium
Halse et al., 2005 [45]	Cross-Sectional Study (prospective)	Australia	TF 1	23 (23)	12–20	Adolescent Medical Ward	Examine the meanings that patients attached to NG	Primary (N/A)	Categories: unpleasant physical experience, a necessary intervention, a physical and psychological signifier of AN, a focus in a struggle for control.	Medium
Clausen et al., 2018 [46]	Cross-Sectional Study (retrospective)	Denmark	TF 13	4727 (4387)	10–40+	Psychiatric/ Medical Ward	Frequency of various involuntary measures in AN patients	Secondary (not discussed)	Involuntary tube feeding was most frequent measure used.	Low
Bayes and Madden, 2011 [47]	Case Series (retrospective)	Australia	TF 2	10 (0)	10–13	Paediatric medical Hospital	Demographic and clinical features of male inpatients with EO ED	Secondary (Medical instability)	Only 3/10 participants met full criteria for AN; 60% required NG feeding.	High
Kodua et al.,2020 [48]	Case Reports (prospective)	UK	TF 1	8 (n/a)	n/a	ED inpatient units	Nursing assistants’ experiences of manual restraint for NG feeding	Primary (N/A)	3 primary themes were gathered: an unpleasant practice, importance of coping, becoming (de)sensitized to NG feeding.	High

Key: *N* Number of participants, *FU* Follow up, *TF* Time Frame, *NG* Nasogastric (Feeding), *LOS* Length of Stay, *ED* Eating disorder, *EO* Early onset, *AN* Anorexia nervosa, *RS* Refeeding syndrome, *%IBW* Percentage ideal bodyweight, *HP* Hypophosphataemia, *OR* Oral refeeding, *RCT* Randomised control trial

## Results

### Prevalence and epidemiology

YP with ED requiring NG were often medically unstable on admission [9, 18, 20, 22, 23, 25, 39] and NG feeding was implemented as standard practice [22, 23, 26, 39]. NG was also implemented due to acute refusal of food or inability to meet oral intake, without significant medical instability, in five studies [9, 10, 18, 31, 43]. In 13 studies (3 high risk of bias [28, 35, 47]) in which NG was not implemented as standard protocol for all patients, the percentage of ED YP administered NG feeding in all contexts (due to medical instability or inadequate oral diet) varied between 6 and 66% [9, 18, 29, 31, 36, 37, 41–44].

Two studies [37, 43], found NG feeding was more likely to be required in: patients of a lower age at admission (14.3 years compared to 15.3 yrs. old, *P* < 0.05 [37] and 20% in early onset AN compared to 0% in adult onset AN P < 0.05 [43]). Clausen [46] described NG as the most frequently used involuntary measure in psychiatric practice and is most commonly used in 15–17 year olds. Studies included both male and female patients, however, out of 25 patient focused studies, most had a female majority and 6 studies [20, 26, 37, 39, 43, 44] were conducted on female only cohorts. 2 studies [21, 47] examined male only cohorts but both were high risk of bias. 1 study [39] included only Caucasian participants however the majority of studies were conducted in affluent, Caucasian majority countries; 31% of the studies included were set in Australia, 14% in the USA, 10% in Canada. There were no studies from Asia, South America or Africa. In Australian based studies, NG was given due to refusal of oral intake in 2 studies [9, 10] as well as to treat medical instability [26]. Globally studies from North America [18, 21, 39, 41] and Turkey [36] focused on medical instability in YP with ED. In the UK, three studies described NG use during medical instability after oral intake was refused [27, 28, 40] and one where oral intake was inadequate [31].

### Reported weight gain

Four studies reported weight gain primarily in the context of ED YP with medical instability [24–26, 44]. 2 of these studies [24, 26] for the first 24–72 h started with continuous NG feeding, using higher than standard calorie protocols, 2400–3000 kcal per day prevented any initial drop in weight. Between admission and discharge, Parker et al [24] reported a mean overall weight gain of 7.4kgs, Kezelman 2018 [26] reported a mean overall increase of 3.04 kg/m^2^ BMI; Madden et al [25] reported a mean weight gain of 2.79 kgs during medical instability using continuous NG feeding at 2400 kcals per day. Skrik Liever et al [44] reported 27% required NG feeding and linked this to a faster weight gain but gave no information related to NG feeding protocols.

Three studies reported weight gain in the context of inadequate oral intake [9, 18, 39]. Maginot et al., 2017 [18] and Whitelaw et al., 2010 [9] reported NG bolus feeding in 13.8 and 15% in order to supplement oral diet with a mean weight gain of 3.1kgs and 2.6kgs respectively but did not report if this was specific to NG feeding. Robb et al [39] compared nocturnal NG feeding to supplement oral diet (maximum 3255 kcals /d) with oral intake (max 2508 kcals/d) reporting nocturnal NG feeding weight gain of 5.4kgs versus 2.4kgs in the oral diet only group.

One study reported on weight gain where NG is routinely started on all ED YP regardless of context [23]. Agostino et al [23] compared a higher calorie (1500-1800 kcal/d) continuous NG fed cohort to lower calorie oral bolus cohort (1000-1200 kcal/d, divided 6 times per day), results showed mean weight gain was greater in the continuous NG fed group (1.22 kgs per week) than the oral bolus fed group (0.08 kgs per week) over the first 2 weeks.

### Patient and staff experience of nasogastric feeding

Five studies used qualitative methods to analyse patient, parent and professional opinions on NG feeding [10, 20, 40, 45, 48]. A survey of dietitians found 82% considered NG feeding a necessary procedure if oral diet is inadequate [10]. Psychiatric nursing assistant’s views centred around: NG being an unpleasant practice, becoming sensitized or desensitized, and the importance of developing coping mechanisms to manage the distress.

An Australian study [45] (conducted in a paediatric unit) found YP viewed being NG fed as: an unpleasant experience, a necessary intervention, a psychological signifier of illness, and an emphasis in an underlying struggle for control. Some described NG feeds as easier than eating as it “disguised” the amount due to not swallowing; others felt it was a form of punishment for not gaining enough weight. Conversely the YP in Paccagnella and colleagues [20] research stated NG was helpful, particularly initially when an oral diet was challenging to manage.

### Feeding regime and calorie intake

A variety of different feeding regimes were identified in this review which are summarised in Table 2. Refeeding protocols daily calorie intake varied greatly between studies particularly as many studies were evaluating the outcome of higher calorie refeeding protocols [9, 18, 22, 24, 31]. Most studies tailored the calorie requirements to the individual patient, accounting for initial weight for height percentage and signs of medical instability. The majority commenced on daily intake of less than 2000 kcal and increased periodically.

**Table 2:** Nasogastric Feeding Protocol and Complications Identified in Studies Included in this Systematic Review

Study	Risk of Bias	Setting	Method and Reason for Implementation of NG	Feeding Regime	Complications
**Whitelaw et al, 2010** [9]	Medium	Medical Ward	Oral intake supplemented with bolus NG feeding if oral RDI not met	Minimum of 1900kcals on day 1 and increased by 300 kcal per day	38% developed HP. HP was associated with lower %IBW on admission
**Rocks et al, 2014** [10]	Medium	MH and Medical Wards	High energy supplements and NG feeds were commonly used to meet RDI.	The initial calorie intake recommended was between 800-1750kcals	Not discussed
**Maginot et al, 2017** [18]	Medium	Medical Ward	Bolus NG feeds supplemental to oral intake if RDI not met	Average of 1185 kcal average which increased to an average of 1781 kcals (range 1500–3000 kcals)	Hypomagnaemia and HP reported, HP was more likely in those under 80% %IBW
**Paccagnella et al, 2006** [20]	Medium	Unknown	Continuous NG feeding until medically stable	15.9–19.7 kcal/kg/day; increased to 30 kcal/kg/day after 24 h.	No patient developed nausea, vomiting, or worsened abdominal symptoms; 2 developed lower limb oedema despite slow infusion.
**Silber et al, 2004** [21]	High	MH Ward	Routine nocturnal NG feeding to supplement daily oral intake vs oral refeeding only (control)	Nocturnal NG feeding regime patients were prescribed calories individually (max 4350 kcal) and 3400 in the oral refeeding group (control).	Epistaxis, nasal irritation.
**Madden et al, 2015** [22]	Low	Medical Ward	Continuous NG feeding until medically stable; followed by oral intake with supplemental nocturnal NG feeding until biomarkers stabilised.	NG feeding continuously for 1–2 days. Weight gain aim for 1 kg per week. Weaning to oral diet occurred as soon as medically stable – average 14 days on NG with feed of 2400-3000 kcal per day	Not discussed
**Agostino et al, 2013** [23]	Medium	Medical Ward	Routine continuous NG feeding at a higher calorie intake compared to lower calorie standard oral intake.	Starting range for NG cohort 1200-2000 kcal increased by 200 kcal/day vs. oral diet of 800-1200 kcal increased by 150 kcal/day. NG fed for 7 days then weaned over 3 days with kcal via NG reducing as meals replaced	Oral cohort 51% lost weight initially compared to 6% in the NG high kcal cohort. 2 cases of Hypokalaemia (although both were abusing laxatives), HP.
**Parker et al, 2016** [24]	Medium	MH Ward	Continuous NG feeding or combination of oral intake with supplemental overnight NG feeding, or oral intake alone.	Start feed 2400 kcal increasing to 2400-3400 kcal/day at 100 ml per hour	Peripheral oedema (4%), hypomagnaemia (7%), hypokalaemia (2%), HP (1%). No incidence of RS or delirium.
**Madden et al, 2015** [25]	Low	Medical Ward	Continuous NG feeding until medically stable; followed by oral intake with supplemental nocturnal NG feeding until biomarkers stabilised. Average %IBW at initiation was 78	2400-3000 kcal to meet weekly target of weight gain of 1 kg/week. In the first week average weight gain was 2.79 kg.	Stated none developed RS or HP
**Kezelman et al 2018** [26]	Medium	Medical Ward	Continuous NG until medically stable followed by oral intake supplemented by nocturnal NG feeding	2400 kcal/day for 24 h or until medically stable, changed to oral diet starting ~ 1800 kcal increasing to a maximum of 3800 kcal with nocturnal NG top up feeds stopped when BMI > 18.5	Not discussed
**Fuller et al, 2019** [27]	Medium	MH Ward	Results from questionnaire showed non-specialist psychiatric units gave 73% NG as syringe bolus, 27% as enteral pump. Specialist ED units gave 85% as syringe bolus, 15% as enteral pump.	Volume of bolus feed ranged from 330 to 1000 ml average 564 ml per feed. Bolus feed time ranged between 10 and 40 min average being 20 min. If delivered by pump it was > 1 h.	Not discussed
**Street et al, 2016** [28]	High	Medical Ward	Bolus NG feeding if medically unstable and oral intake not met	NG feeds were higher in calories than meals to motivate eating.	Not discussed
**Couturier and Mahmood, 2009** [29]	Medium	MH Ward	Bolus NG feeding if patient failed to gain 1 kg/week or acute refusal of meals	Not discussed	Nausea, odynophagia, self-harm, epistaxis, anxiety, sadness, 38.4% patients experienced mild HP
**Falcoski et al, 2020** [30]	High	MH Ward	Oral calories supplemented with bolus NG feeds, single bolus of high calorie NG feeding, and 3 smaller single boluses.	Starting feed 1200 kcal, increased by 200 kcal per day to 2000 kcal. 1 NG feed per day under restraint. Also described 1 bolus feed of 2000 kcal due to no oral intake for 20 h	Distress described during the procedure requiring Lorazepam
**O’Connor et al, 2016** [31]	Low	Medical Ward	Supplemental bolus NG feeding if patients failed to meet 80% RDI. At initiation %IBW was < 78%	Compared 500 kcal starting diet with 1200 kcal	HP (28%)
**Akgul et al, 2016** [35]	High	MH Ward	Not discussed	Initiated at 750 kcal per day and increased by 220 kcal per day	HP described in 2 cases (unable to determine if this was in those requiring NG)
**Akgul et al, 2016** [36]	Medium	Medical Ward	Not discussed, the majority of young people were under 80% %IBW	Started on an average of 975 kcal. Average duration of NG was 2.5 days	HP described in 2 cases (not stated if this was in those requiring NG)
**Robb et al, 2002** [39]	Medium	Medical Ward	Nocturnal NG feeding to supplement daily oral intake during medical instability	Starting NG feed at 600 kcal. Ratio oral kcal to NG was approximately 2:1. NG feed via pump at 40 cc per hour for 4 h then 60 cc per hour for 4 h. Increases to 1200 kcal NG feed over 3 nights. Weaned when the young person is 95%IBW.	Epistaxis (11.5%), anxiety (3.8%) treated with Lorazepam, removal of NG tube (5.8%), nasal irritation (28.8%).
**Neiderman et al, 2001** [40]	High	Medical Ward	Not discussed	Not discussed	Removal of tube (55%).

Key: *BMI* Body Mass Index, *NG* Nasogastric, *MH* Mental health, *RDI* Recommended daily intake, *HP* Hypophosphataemia, *RS* Refeeding syndrome, *%IBW* Percentage ideal bodyweight

No study discussed in detail the strategy used to transition from NG feeds back to an oral diet. Those studies where NG was used for medical stabilisation often described a short period of NG before a quick transition back to an oral diet [22, 23, 36]. In studies where continuous NG was provided, YP were sometimes not given the option of an oral diet so that their calorie intake could be closely monitored [22–24, 31]. These studies discussed ceasing NG feeds after the risk of RS had reduced; most gave a time frame between 2 and 14 days [24, 44]. Studies using bolus feeds stated that oral intake was encouraged and it was only when this was not fully achieved that supplementary NG was used [39]. This appeared to be either after each meal, at set times during the day or once in the evening [27]. For nocturnal feeds, oral diet was encouraged during the day. In most studies the NG feed supplemented any deficit in oral intake but occasionally also provided additional calories above those prescribed in the oral meal plan [22, 25, 39].

### Length of time receiving NG feeding

There was a wide variety in length of time receiving NG for medical instability. Agostino and colleagues [23] delivered nutrition on a medical ward solely via NG for 14 days before commencing oral diet in addition to NG feeding. The average length of time on NG feeding in this study was 20.7 days; NG was terminated as YP accepted more than 50% oral caloric quota compared to theoretical reported quota. Madden et al [22] RCT determined the duration of NG feeding was a minimum of 14 days, using biochemical markers of medical instability in a hospital setting. Conversely, Akgul and colleagues [36] described a much shorter average time, 2.5 days, that YP required NG before transitioning to an oral diet. Conversely, in MH wards, if NG has to be given under restraint, it may be required for a significant duration; in one study [46] the average was 170 days. Neiderman et al [40] qualitative study describes patients time receiving NG varying from 1 to 476 days (methods not explained).

Two studies examined therapeutic interventions to reduce the need for NG or length of time on it in medically stable YP [29, 41]. Couturier and Mahmood [29] highlighted that meal support therapy reduced the requirement for NG feeding from 66.7 to 11.1%, criteria for NG feeding was the same in both groups throughout and oral intake was encouraged. Gusella and colleagues [41] compared parent led therapy (PLT) to non-specific therapy (psychologist led talking therapy). PLT was based on FBT and included parents reducing child exercise and increasing oral intake. Results demonstrated that YP receiving PLT had a significantly reduced requirement for NG (*P* < 0.05).

### Complications associated with NG feeding

Complications associated with NG feeding found in this review are summarised in Table 2, with the most frequently described being nasal irritation or epistaxis, anxiety related to the procedure and electrolyte disturbance (which occurred with both oral and NG refeeding). Overall, this review found 5 studies [9, 18, 23, 24, 29] reported some incidence of electrolyte disturbance, 2 studies [29, 39] described epistaxis and 1 study [39] described behavioural problems associated with the procedure. A number of YP in MH wards required restraint to NG feed with one study reporting this was required for 66% of YP [24]. NG under restraint was described as causing distress and risk of injury to both staff and YP [48]. No study reported a YP developed RS. Nehring and colleagues [37] concluded that NG feeding had no impact on growth, recovery or development of psychiatric co-morbidities.

Kezelman and colleagues (Australia) 2018 [26] assessed the impact on anxiety, depression and ED symptoms when using NG in adjunct to oral intake as part of a rapid refeeding regime. Changes in these symptoms were not attributed to the rate of weight restoration suggesting a rapid refeeding schedule would not exacerbate psychiatric symptoms.

### Length of stay associated with NG feeding

Length of stay was reported in studies from medical and MH ward settings, however, the specific package of treatment YP received in each study was different depending on the country of origin. For example, in Australian studies medical wards tended to include high levels of psychiatric treatment alongside medical treatment [26]. Agostino and colleagues [23] demonstrated that YP on medical wards having NG feeds had a mean LOS of 33.8 days compared to those in the same setting having an oral diet who had a mean of 50.9 days, however, the oral diet was lower in calories therefore taking longer for weight recovery and medical stabilisation. Conversely any hospital admission was significantly longer (*P* < 0.0001) for a YP requiring NG feeding compared to those managing an oral diet in a German retrospective cohort study [37]. However, this study does not discuss the reasons NG was implemented. Maginot et al. study [18] in a medical ward (where NG was implemented due to insufficient oral intake) discussed NG feeding in the context of YP with more severe medical problems, (such as intractable vomiting and superior mesenteric artery syndrome) which therefore took longer to transition to oral diet, resulting in a longer admission.

Strik Lievers and colleagues [44] concluded that, amongst others, requirement for NG feeding when NG was implemented due to medical instability was a factor affecting LOS on a psychiatric ward. In this study the mean LOS was significantly increased: 117 days for YP managing oral intake compared to 180 days for those requiring NG. They concluded that the requirement for NG was an indication of severity and resistance to oral feeding [44].

## Discussion

It is evident that there is a wide variety of practices regarding implementation and regime of NG feeding in YP with eating disorders globally [9]. Given that the procedure can be painful [48] for YP and cause complications [29, 39], there is an urgent need for research exploring this wide variation in use of NG feeding to enable future direction and best practice guidance clinicians. A review conducted by Rizzo and colleagues [49] (2019), which focused on NG for acute refeeding, also found a wide variety of practices.

From this systematic review 3 methods of NG feeding in YP with ED were found: continuous [23, 25], nocturnal [26, 29], and bolus meal replacement [9]. It is not possible from this review to discern the advantages and disadvantages of each method as no study made a direct comparison. When NG feeding is used under restraint bolus feeds are preferred due to concerns around the tube being removed by the YP once restraint had ceased [45]. The main disadvantage to bolus feeding, in medically stable YP, is that the NG tube requires reinsertion each time a feed is required, however, it provides a tangible motivation to eat the full meal plan provided which, in practice, should always be encouraged over NG feeding in order to promote patient wellbeing. Further research is required to assess which method is the safest, most efficacious and best aids transition back to a fully oral diet.

Medical wards used continuous feeding more frequently than MH wards, however this tended to be for a short period of time while the YP was medically unstable, after this they would be transitioned to an oral diet [22, 23, 25, 26]. It is probable that medical wards primarily manage YP for short periods to stabilise acute physical health deterioration, while MH wards admit relatively medically stable YP and seek primarily to treat psychological ED symptoms that are preventing an adequate oral diet. This difference could account for the divergent outcomes from studies on the impact NG has on the LOS between medical and psychiatric settings [23, 44].

Similar to the review conducted by Hale and Logomarsino [33] who found RS to be a rare complication, it is reassuring to find that no study in this review reported YP developing RS despite some studies starting on high calorie NG feeding plans [9, 18, 24, 42]. Although complications such as electrolyte abnormalities did occur there was no evidence that this was attributable to the NG feeding compared to oral diet [9, 18, 23, 24]. The results of this review support the conclusions from Rizzo and colleagues [49] (2019) that NG feeds can be safely administered and have the advantage of shortening LOS when used to increase total caloric intake. However, further research is required to assess the optimum NG feeding regime for YP at different levels of RS risk. In two studies intensive meal support and concurrent therapy reduced the number of NG episodes (in medically stable YP) before managing a full oral diet [29, 41]. This could have the advantage of reducing LOS in medically stable YP.

There are a number of limitations to the conclusions that can be drawn from this review. The majority of studies included were retrospective and based around case note reviews which are subjective and therefore likely to be biased. A retrospective design also creates selection bias as those lost to follow up are not considered. Bias can also occur due to the different treatment groups being recorded at different times thus confounding variables may include different staff working at the setting and therefore different methods of treating YP. Only 52% of studies were conducted prospectively. Three studies were qualitative interview studies, examining patient or staff feelings towards NG feeding in practice which increases the risk of confirmation bias. The majority also had a relatively small sample size, again introducing the possibility of bias and reducing generalizability. 58% of the studies included only examined the effect of NG feeding as a secondary outcome of their study. It is not possible from these studies to make any comparison between NG feeding and oral intake due to the confounding effect that for the vast majority of studies only high risk, medically unstable YP were considered for NG feeding. Pragmatic, prospective studies that control for this confounder are required for any such comparison to be made.

## Conclusions

This review describes the large differences in the use of NG for YP with ED in medical and psychiatric wards in a number of countries globally. NG feeding is an important aspect of treatment for YP with ED who are medically unstable and/or unable to manage an adequate oral diet. Although there are some RCT’s examining aspects of NG use in YP with ED the majority of studies were retrospective cohorts or case series. There is a need for more high quality data in when to initiate NG, comparing different methods of delivering NG feeds and transitioning from NG to oral diet in YP with restrictive ED to enable future direction for clinicians.

### Supplementary Information

**Additional file 1: Appendix 2.** Risk of bias in eligible studies.

## Data Availability

All articles analysed in this study can be found in Table 1 and can be traced back to primary articles using References on Page 16.

## Appendix 1

1. Naso-gastric or nasogastric or *enteric or *enteral or tube
2. (Anorexia or bulimia or eat* or feed*) NOT bowel NOT surgery NOT intestin*
3. (child* or paed* or adolescen* or teen* or young) NOT baby NOT toddler NOT infant NOT animal NOT maternal NOT parental NOT learning disabl* NOT learning disabil*
4. 1 AND 2 AND 3

## Abbreviations

%IBW: Percentage ideal body weight
AN: Anorexia nervosa
AOED: Adolescent onset eating disorder
CAMHS: Child and adolescent mental health service
ED: Eating disorder
EOED: Early onset eating disorder
FBT: Family-based therapy
HP: Hypophosphataemia
LOS: Length of stay
MH: Mental health
NG: Nasogastric feeding
OT: Occupational therapist
PLT: Parent led therapy
PRISMA: Preferred Reporting Items for Systematic Reviews and Meta-Analyses
RDI: Recommended daily intake
RS: Refeeding syndrome
YP: Young person/people
