# Supplementary material for: A systematic review of enteral feeding by nasogastric tube in young people with eating disorders
Source: J Eat Disord. 2021 Jul 22;9:90. doi: 10.1186/s40337-021-00445-1 (PMC8299614; doi:10.1186/s40337-021-00445-1)
Supplement: Supplementary file 1 — Additional file 1: Appendix 2. Risk of bias in eligible studies. [file 40337_2021_445_MOESM1_ESM.docx]

**Appendix 2** Risk of Bias in eligible studies

| **Authors** | **Study design** | **Sample size** | **Unbiased cohort selection** | **Selection minimizes baseline differences in demographic factors** | **Sample size calculated** | **Validated method for ascertaining clinical status or participant group** | **Validated methods for assessing variables of interest** | **Validated methods for assessing outcome** | **Blind outcome assessment** | **Score** |
| --- | --- | --- | --- | --- | --- | --- | --- | --- | --- | --- |
| Whitelaw et al., 2010^9^ | 2 | 2 | 0 | 0 | 0 | 6 | 6 | 6 | 0 | 22 |
| Rocks et al., 2014^10^ | 4 | 2 | 2 | 2 | 0 | 6 | 6 | 6 | 0 | 28 |
| Maginot et al., 2017 ^18^ | 2 | 4 | 2 | 2 | 0 | 6 | 6 | 4 | 0 | 26 |
| Paccagnella et al., 2006^20^ | 4 | 2 | 0 | 2 | 0 | 6 | 6 | 6 | 0 | 26 |
| Silber et al., 2004^21^ | 2 | 2 | 0 | 0 | 0 | 6 | 6 | 4 | 0 | 20 |
| Madden et al., 2015b^22^ | 6 | 4 | 4 | 4 | 0 | 6 | 6 | 6 | 6 | 42 |
| Agostino et al., 2013^23^ | 2 | 6 | 0 | 0 | 6 | 6 | 6 | 6 | 6 | 38 |
| Parker et al., 2016^24^ | 2 | 6 | 6 | 0 | 0 | 6 | 6 | 6 | 0 | 32 |
| Madden et al., 2015a^25^ | 6 | 4 | 6 | 4 | 6 | 6 | 6 | 6 | 6 | 50 |
| Kezelman et al 2018^26^ | 4 | 2 | 2 | 2 | 0 | 6 | 6 | 6 | 0 | 28 |
| Fuller et al., 2019^27^ | 4 | 6 | 6 | 6 | 0 | 6 | 6 | 6 | 0 | 40 |
| Street et al., 2016^28^ | 0 | 2 | 0 | 0 | 0 | 0 | 0 | 6 | 0 | 8 |
| Couturier and Mahmood, 2009^29^ | 2 | 2 | 0 | 0 | 0 | 6 | 6 | 6 | 0 | 22 |
| O'Connor et al., 2016^31^ | 6 | 2 | 4 | 4 | 6 | 6 | 6 | 6 | 6 | 46 |
| Falcoski et al.2020^30^ | 0 | 0 | 0 | 0 | 0 | 6 | 6 | 6 | 0 | 18 |
| Akgul et al., 2016a^35^ | 0 | 2 | 0 | 0 | 0 | 6 | 0 | 0 | 0 | 8 |
| Akgul et al., 2016b^36^ | 2 | 2 | 0 | 0 | 0 | 6 | 6 | 6 | 0 | 22 |
| Nehring et al., 2014^37^ | 2 | 6 | 4 | 2 | 6 | 6 | 6 | 6 | 0 | 38 |
| Neiderman et al., 2000^38^ | 0 | 0 | 0 | 0 | 0 | 6 | 6 | 4 | 0 | 16 |
| Robb et al., 2002^39^ | 4 | 6 | 4 | 4 | 0 | 6 | 6 | 6 | 0 | 36 |
| Neiderman et al., 2001^40^ | 4 | 2 | 0 | 0 | 0 | 6 | 0 | 0 | 0 | 12 |
| Gusella et al., 2017^41^ | 2 | 2 | 0 | 0 | 0 | 6 | 6 | 6 | 0 | 22 |
| Madden et al., 2009^42^ | 4 | 6 | 6 | 6 | 0 | 6 | 0 | 0 | 0 | 28 |
| van Noort et al., 2018^43^ | 4 | 6 | 4 | 4 | 0 | 6 | 6 | 6 | 0 | 36 |
| Strik Lievers et al., 2009^44^ | 4 | 6 | 4 | 4 | 0 | 6 | 6 | 6 | 0 | 36 |
| Halse et al., 2005^45^ | 4 | 2 | 2 | 2 | 0 | 6 | 6 | 6 | 0 | 28 |
| Clausen et al., 2018^46^ | 4 | 6 | 6 | 6 | 2 | 6 | 6 | 6 | 0 | 42 |
| Bayes and Madden, 2011^47^ | 0 | 2 | 2 | 0 | 0 | 6 | 2 | 4 | 0 | 16 |
| Kodua et al.2020^48^ | 0 | 0 | 0 | 0 | 0 | 6 | 6 | 6 | 0 | 18 |

Key: Study design: RCT=6 Prospective/cross section=4 Retrospective cohort=2 Case series=0 Sample size: >100=6, 50-100=4, 10-50=2, <10=0. Other columns: Clear evidence =6 Some evidence =4 Little evidence=2 No evidence =0., Total score: 0-20= High risk, 21-40= Medium risk >40=Low risk
